# Breast neuroendocrine tumor arising in the axilla of a man: a case report

**DOI:** 10.1186/s13256-022-03683-2

**Published:** 2022-12-18

**Authors:** Kosei Kimura, Shigeru Kawabata, Hiroyo Oku, Ayana Ikari, Tomo Tominaga, Saki Takai, Junna Sakane, Michiaki Tanaka, Chinatsu Aoki, Monika Ota, Erika Minami, Yoshinobu Hirose, Sang-Woong Lee, Mitsuhiko Iwamoto

**Affiliations:** 1 Department of Breast and Endocrine Surgery, Osaka Medical and Pharmaceutical University, Osaka, Japan; 2 Department of Pathology, Osaka Medical and Pharmaceutical University, Osaka, Japan

**Keywords:** Accessory breast carcinoma, Neuroendocrine tumor, Male breast cancer, Axillary tumor

## Abstract

**Background:**

Accessory breast carcinomas of the axilla of males are rare, and primary breast neuroendocrine tumors (BNETs) are rare as well. We present a case of a BNET arising in the axilla of a man.

**Case presentation:**

A 64-year-old Japanese man presented with a hard 15-mm mass in the axilla and axillary lymph node swelling. Histopathological examination of the incisional biopsy specimen revealed a neuroendocrine carcinoma. Therefore, wide radical excision of the axillary tumor and axillary lymph node dissection were performed. Hematoxylin and eosin staining showed that the solid tumor was mainly located in the subcutaneous adipose tissues and appeared to invade the skin. The tumor phenotypes were positive for CAM 5.2, synaptophysin, estrogen receptor, progesterone receptor, and GATA-binding protein 3; they were negative for human epidermal growth receptor 2. The neuroendocrine component comprised more than 90% of the tumor, and the Ki-67 index was 21%. These results indicated that the tumor was a BNET. This patient underwent adjuvant chemotherapy, endocrine therapy, and radiotherapy.

**Conclusions:**

BNET cases in males are rare. The clinical and histological criteria as well as treatment for these rare cases are discussed.

## Background

Adenocarcinomas of the axilla of males are rare and may include sebaceous or sweat gland cancer, accessory breast cancer, or metastasis from an unknown primary tumor [1–3]. Neuroendocrine tumors (NETs) are neoplasms that arise from the neuroendocrine cells and can occur anywhere in the body. The prevalent site is the gut, but the mammary gland is uncommon [4]. We present a case of a primary breast NET (BNET) arising in the axilla of a man because such cases are extremely rare and often difficult to diagnose and treat.

## Case presentation

**Fig. 1:**
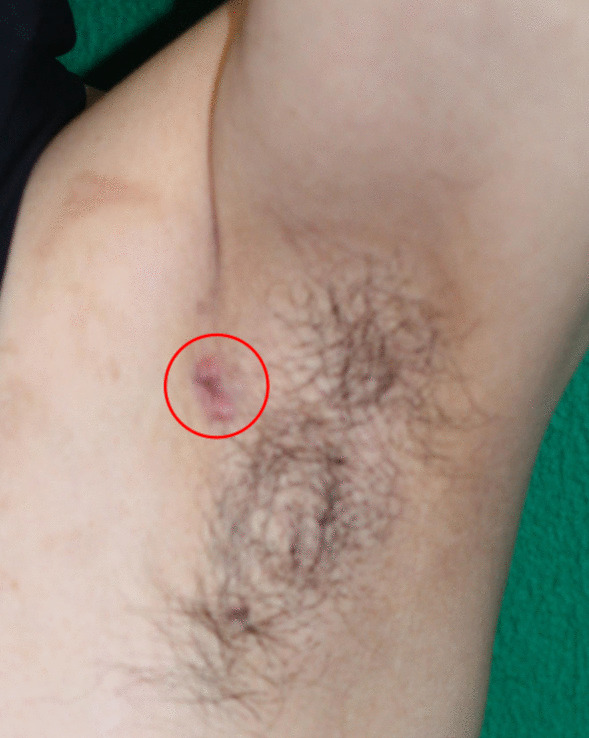
An irregular hard mass measuring 15 × 10 mm in the patient’s left axilla (red circle)

**Fig. 2:**
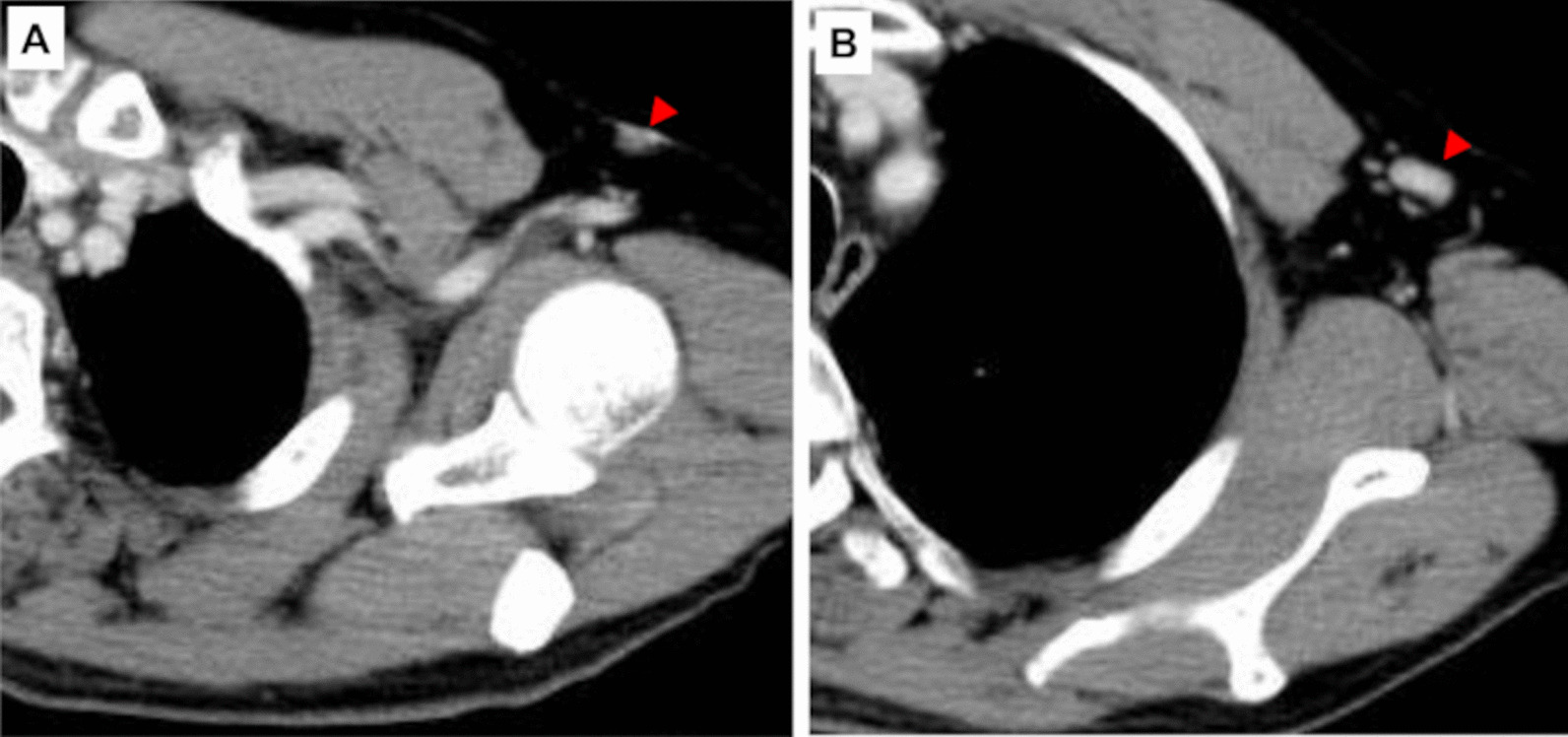
Computed tomography (CT). **A** CT shows an irregular mass in the left axilla (red arrowhead). **B** Some enlarged axillary lymph nodes are seen (red arrowhead)

A 64-year-old Japanese man became aware of a small subcutaneous nodule in his left axillary region in 2019. The nodule was progressively enlarging; therefore, the patient presented to a dermatologist in 2021. He had no notable medical history or family history of disease. He had also no history of alcohol consumption, no history of smoking, no regular medications. The findings of an incisional biopsy of the nodule confirmed the diagnosis of neuroendocrine carcinoma; therefore, he was referred to our hospital. On palpation, an irregular, hard mass with a measurement of 15 mm × 10 mm was found in the left axilla (Fig. 1), but no other abnormalities on physical or neurological examination. Laboratory tests and vital signs also showed no abnormality. Ultrasonography revealed a 15-mm irregular mass in the axilla with enlarged lymph nodes, but the bilateral mammary glands appeared normal. Computed tomography (CT) showed an irregular mass in the left axilla with enlarged axillary lymph nodes (Fig. 2A, B). No evidence of distant metastasis was observed. Additionally, fluorodeoxyglucose (FDG)-positron emission tomography (PET)/CT revealed no significant findings other than the axillary tumor. Therefore, wide radical excision of the axillary tumor and axillary lymph node dissection were performed.

**Fig. 3:**
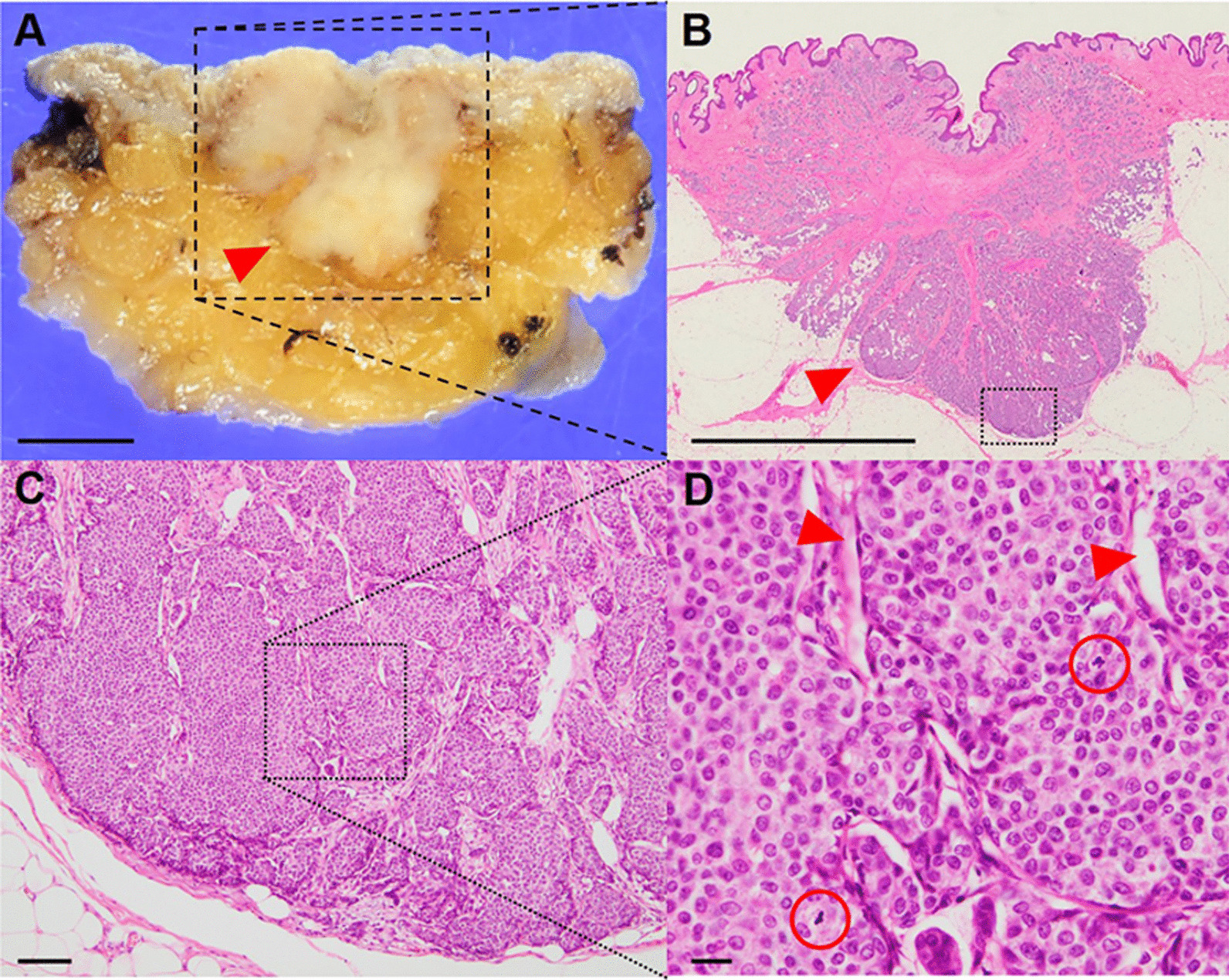
An invasive tumor located in the skin and subcutaneous adipose tissues. **A** Macroscopic features of a sliced surface. The red arrowhead indicates a nodular lesion. Scale bar, 5 mm. **B** Hematoxylin and eosin section of an invasive tumor (red arrowhead), shown in the black dotted lined inset in panel A. Loupe image; scale bar: 5 mm. **C** The tumor consists of solid nests, shown in the magnification of the black dotted lined inset in panel B. Magnification, 100 × ; scale bar, 100 μm. **D** Densely cellular and solid nests separated by fibrovascular cores (red arrowhead), shown in the magnification of the black dotted lined inset in **C**. Red circles: mitoses; Magnification, 400 × ; scale bar, 20 μm

**Fig. 4:**
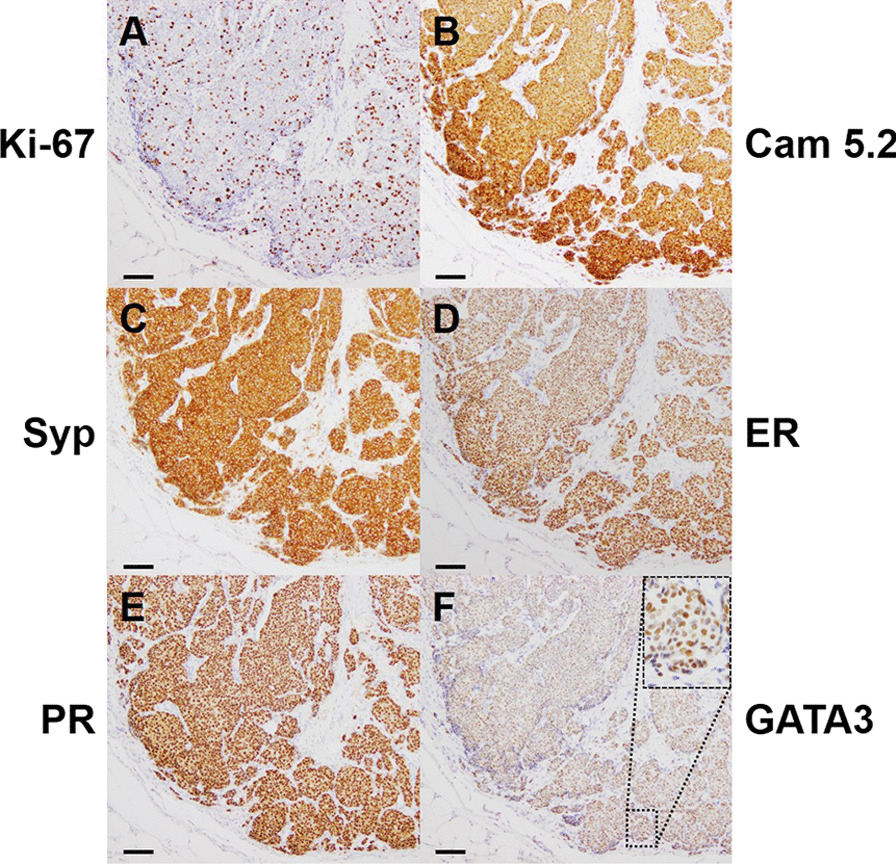
Proliferation and phenotypes in the tumor. **A** Ki-67 staining; the proliferation index was 20.8% in the densest spot of the high-power field (400 × magnification) and was calculated using the following formula: ([the number of positive tumor cells/total number of tumor cells] × 100). **B**–**F** Phenotypes in the tumor assessed with immunohistochemical analyses for Cam 5.2 (**B**), synaptophysin (Syp: **C**), estrogen receptor (ER: **D**), progesterone receptor (PR: **E**), and GATA-binding protein 3 (GATA3: **F**). A-F. Magnification, 100 × ; scale bar, 100 μm

Macroscopically, the sliced surface of the formalin-fixed tissue revealed a yellow-white nodular lesion on the skin and subcutaneous adipose tissue with a measurement of 15 × 14 × 11 mm (Fig. 3A). Hematoxylin and eosin (H&E) staining showed that the solid tumor was mainly located in the subcutaneous adipose tissue and appeared to invade the skin (Fig. 3B). The tumor consisted of dense cellular solid nests separated by fibrovascular cores. Cellular uniformity, ovoid nuclei with salt-and-pepper chromatin, and eosinophilic cytoplasm were also observed. The number of cells undergoing mitosis was nine per ten high-power fields (Fig. 3C, D). The Ki-67-positive cells increased, with a proliferation index was 20.8% (Fig. 4A). The tumor phenotypes were strongly positive for CAM 5.2, which is a cytokeratin (CK) marker, and synaptophysin (Syp), which is a neuroendocrine marker, indicating that the tumor was an epithelial neoplasm with neuroendocrine differentiation (Fig. 4B, C). The neuroendocrine component comprised more than 90% of the tumor. Additionally, the tumor was positive for estrogen receptor (ER) and progesterone receptor (PR) (Fig. 4D, E), suggesting that it was likely derived from the mammary gland; this was supported by GATA-binding protein 3 (GATA3)-positive staining (Fig. 4F). Additionally, the tumor was negative for human epidermal growth receptor 2 (HER2). Therefore, these findings indicated that the tumor was a BNET. Three lymph nodes were histologically metastatic; therefore, this patient was diagnosed with the equivalent of pT1N1M0 stage IIA conventional breast carcinoma.

The patient received four courses of Taxotere and cyclophosphamide (TC) chemotherapy (75 mg/m^2^ docetaxel and 600 mg/m^2^ cyclophosphamide) every 3 weeks. Endocrine therapy (20 mg/day tamoxifen) and radiotherapy of the left breast and supraclavicular lymph nodes (50 Gy/25 Fr) were started after chemotherapy. One year has passed since the surgery, and there has been no recurrence.

## Discussion

In this report, we present a case of a BNET arising in the axilla of a man. Male accessory breast cancer in the axilla is rare, but BNETs like this case are even rarer. Although the tumor is relatively small (15 mm in size), it already has had the axillary lymph nodes metastasis. Therefore, systemic treatment is considered necessary, but because the disease is rare, the diagnosis and treatment plan is interesting but confusing.

Male breast cancer is comprising approximately 1% of all breast cancer cases [5]. It is often detected as an accessory breast cancer, which usually presents as an axillary tumor [6, 7]. The accessory breast cancer must be pathologically proven to be a solitary lesion adjacent to a normal breast duct or lobule. Additionally, the possibility of metastatic disease from another primary cancer must be excluded [8].

During the diagnosis of our case, it was difficult to differentiate the cutaneous adnexal tumor or metastatic lesion from another primary cancer. Histopathological and immunohistochemistry (IHC) findings revealed features of endocrine mucin-producing sweat gland carcinoma (EMPSGC) that were compatible with the tumor in this case. However, the possibility of EMPSGC was excluded because these tumors are usually found in the head and neck, and they are commonly observed on the eyelids of older women [9]. To rule out a metastatic lesion from another primary cancer, imaging studies, including CT, ultrasonography, and FDG-PET/CT, were performed and revealed no evidence of primary malignancy. However, NETs, especially low-grade NETs, often do not have FDG accumulation, and the use of OctreoScan is recommended to exclude primary NETs at other sites [10]. The pathological examination showed no evidence of normal breast ducts or lobules around the primary lesion; therefore, an examination to determine the presence of NETs at other sites using OctreoScan was considered. However, the immunohistochemical features of the tumor (positive for ER, PR, and GATA3) suggested that this tumor was derived from the mammary gland. Therefore, we diagnosed this axillary tumor as a BNET.

Neuroendocrine neoplasms (NENs) arise from neuroendocrine cells and can occur anywhere in the body. The most common sites include the gut, lungs, and bronchi; however, the soft tissue is an uncommon site [4] and is classified as NETs and neuroendocrine carcinomas (NECs) according to the latest World Health Organization (WHO) edition published in 2019 [11]. Dholaria *et al.* [12] reported a case of NEC in the axilla of a man. IHC of this tumor showed positive Syp, and negative for CK7 and CK20, with a high Ki-67 index (80%). Therefore, he was diagnosed with NEC and axillary metastasis from an unknown primary or de novo [12].

Primary breast NEN (BNEN) is rare, accounting for less than 1% of all NENs, and its definition is confusing [13]. A BNEN is classified breast NEC (BNEC) and BNET by the most recent WHO classification [11]. BNEC is reported to be small cell type and morphologically similar to NEC of other organs [14]. According to the current WHO classification, BNET is considered a mixed neuroendocrine and non-neuroendocrine breast neoplasms with a non-neuroendocrine component of less than 10% [11]. BNEN has a higher expression of ER and PR than other breast cancers and is usually categorized as a luminal A or luminal B HRE2-negative breast cancer [15, 16]. NETs affecting other organs are classified as G1, G2, or G3 based on the mitotic count, Ki-67 proliferation index, and presence of necrosis, whereas BNETs are classified in the same way as nonspecific types of breast cancer [17]. BNETs typically have IHC markers such as CK7, ER, PR, and GATA3 [18]. Our tumor was positive for CAM 5.2, Syp, ER, PR, and GATA3, and it was negative for HER2, with a Ki-67 index of 21%; therefore, we believe that it was a BNET in the luminal A breast carcinoma category.

There are no specific guidelines for the treatment of BNENs; therefore, they are managed with conventional breast cancer treatment. Endocrine therapy has proven effective as an adjuvant therapy for some cases of hormone receptor-positive BNENs, and chemotherapy is widely used for patients at high risk for recurrence [19, 20]. In our case, we diagnosed BNET with three axillary lymph node metastases in a man. The Ki-67 index was 21%, which is equivalent to G3 NET of other organs. The need for postoperative adjuvant treatment is controversial; however, as mentioned, the use of the same treatment used for conventional breast cancer is recommended. Therefore, we administered TC chemotherapy, endocrine therapy, and radiotherapy after surgery.

## Conclusions

We encountered a rare case of BNET in the axilla of a man. There are no specific guidelines for this rare tumor. Therefore, an improved understanding of its pathophysiology is necessary to develop optimal treatment strategies.

## Data Availability

Data sharing does not apply to this article, as no datasets were generated or analyzed during the current study.

## Abbreviations

BNEC: Breast neuroendocrine carcinoma
BNEN: Breast neuroendocrine neoplasm
BNET: Breast neuroendocrine tumor
CK: Cytokeratin
CT: Computed tomography
EMPSGC: Endocrine mucin-producing sweat gland carcinoma
ER: Estrogen receptor
FDG: Fluorodeoxyglucose
GATA3: GATA-binding protein 3
H&E: Hematoxylin and eosin
HER2: Human epidermal growth factor 2
IHC: Immunohistochemistry
NEC: Neuroendocrine carcinoma
NEN: Neuroendocrine neoplasm
NET: Neuroendocrine tumor
PET: Positron emission tomography
PR: Progesterone receptor
Syp: Synaptophysin
WHO: World Health Organization
